# Iodine contrast should be avoided in patients with thyroid eye disease

**DOI:** 10.3389/fopht.2024.1478805

**Published:** 2024-09-27

**Authors:** Jane Z. Spadaro, Brittany A. Simmons, Alon Kahana

**Affiliations:** ^1^ Kahana Oculoplastic and Orbital Surgery, Livonia, MI, United States; ^2^ Beaumont Eye Institute, William Beaumont Hospital, Royal Oak, MI, United States; ^3^ Department of Ophthalmology and Visual Sciences, W. K. Kellogg Eye Center, University of Michigan Medical School, Ann Arbor, MI, United States; ^4^ Department of Ophthalmology, Oakland University William Beaumont School of Medicine, Rochester, MI, United States

**Keywords:** thyroid eye disease, graves’ disease, iodine contrast, orbital inflammation, orbitopathy, graves orbitopathy, computed tomography, iodinated contrast media

## Introduction

Iodinated contrast media (ICM) is known to trigger thyroid dysfunction and thyrotoxicosis in both euthyroid and susceptible individuals (1–3). Despite this, there are few guidelines for screening and management of ICM administration in patients at risk for thyroid dysfunction (4, 5). In addition, the induction and exacerbation of thyroid orbitopathy are frequently overlooked as a sequelae of thyroid dysfunction. As examples of this, in the American Academy of Ophthalmology Basic and Clinical Science Course used by ophthalmology trainees, the computed tomography (CT) scan used to demonstrate the radiographic findings of thyroid eye disease (TED) is one with contrast enhancement (6). In another resource that is widely utilized by ophthalmology trainees and attendings to study for oral boards, Case 84 dictates the use of a CT scan with contrast to evaluate a patient with suspected TED (7). Understanding the pathophysiology of ICM in susceptible individuals as well as its effects on TED will ideally lead to improved patient outcomes.

All iodinated contrast agents contain a tri-iodinated benzene ring (8). The ability of the contrast agent to attenuate x-rays is based upon the number of iodine molecules present and the type of x-ray utilized (8). The most common routes of ICM administration include intravascular, enteric, and direct injection for imaging techniques including, but not limited to computed tomography, fluoroscopy, angiography, and venography. In general, the majority of patients receive 50-100 mL of contrast per CT scan, with higher doses required for more invasive procedures such as cardiac catheterizations and percutaneous coronary interventions (which can range from a fixed volume of 125 mL, a weight-based dose of 3 mL/kg, to a max volume of 300 mL) (2, 9). A typical CT scan provides 2,500 - 5,000 μg of bioavailable free iodine and 15 - 60 gm of total iodine (1, 2, 10). Non-bioavailable iodine can then be liberated to free iodide. Thus iodine stores may remain elevated for up to 4-8 weeks following ICM administration (2, 3). This is in significant excess when compared to the daily recommended iodine intake of 150 μg for an average adult (11).

In the thyroid gland, iodide is actively transported intracellularly into thyroid follicles through the sodium-iodide symporter (NIS), and used for the synthesis of thyroid hormone, thyroxine (T4) and triiodothyronine (T3). With excess iodine exposure, a transient decrease of thyroid hormone production occurs, also known as the Wolff-Chaikoff effect (11). This is due to formation of iodopeptides which inhibit thyroid peroxidase (TPO) activity, an enzyme involved in thyroid hormone production (2). Adaptation or escape from the Wolff-Chaikoff phenomenon occurs as a result of decreased expression of NIS, through downregulation of mRNA and protein production. As a result, less iodide is able to enter into the thyroid follicle (12). The majority of patients return to a euthyroid state within 48 hours to 4 weeks (1, 2).

However, patients with impaired autoregulation are at risk of iodine-induced hyperthyroidism or thyrotoxicosis, also known as the Jod-Basedow phenomenon (2, 10). Known risk factors for iodine-induced thyroid dysfunction include Graves’ disease (even with euthyroid or latent disease), Hashimoto’s thyroiditis, an autonomous thyroid nodule, nontoxic diffuse or nodular goiter, neonates or fetuses, patients with impaired renal function, individuals taking potential goitrogens (e.g. lithium), or a history of one of the following: long-standing iodine deficiency, prior thyroid surgery, subacute or postpartum thyroiditis, Type 2 amiodarone-induced thyrotoxicosis, and/or interferon-alpha therapy (2, 10). Both NIS dysfunction and presence of TPO autoantibodies have been implicated in iodine-induced hyperthyroidism (2). Case control studies have demonstrated an increased risk of thyroid dysfunction up to 9 months post ICM exposure (2, 3).

Thyroid eye disease (TED) is associated with autoimmune thyroid dysfunction, and is a risk factor for iodine-induced thyroid dysfunction (13). There is a growing body of evidence suggesting that higher thyroid antibody titers, which can result from increased thyroid stimulation (e.g. from the Jod-Basedow phenomenon), are associated with more severe and progressive thyroid orbitopathy (14, 15). On the other hand, the presence of thyroid peroxidase or thyroglobulin autoantibodies has been suggested to reduce thyroid dysfunction following ICM exposure (3). While the precise relationship between iodine-induced thyroid dysfunction and thyroid autoantibodies requires further investigation, it is interesting to note that NIS and TPO, enzymes whose dysregulation has been implicated in the Jod-Basedow phenomenon, are expressed in orbital fibrocytes (16). Despite the undetermined effect of excess iodine on orbital fibroblasts, the local effects of ICM on the NIS and autoantibodies targeting orbital tissues may present a mechanistic explanation for susceptible patients who experience an exacerbation of TED following ICM.

## Case series

**Figure 1:**
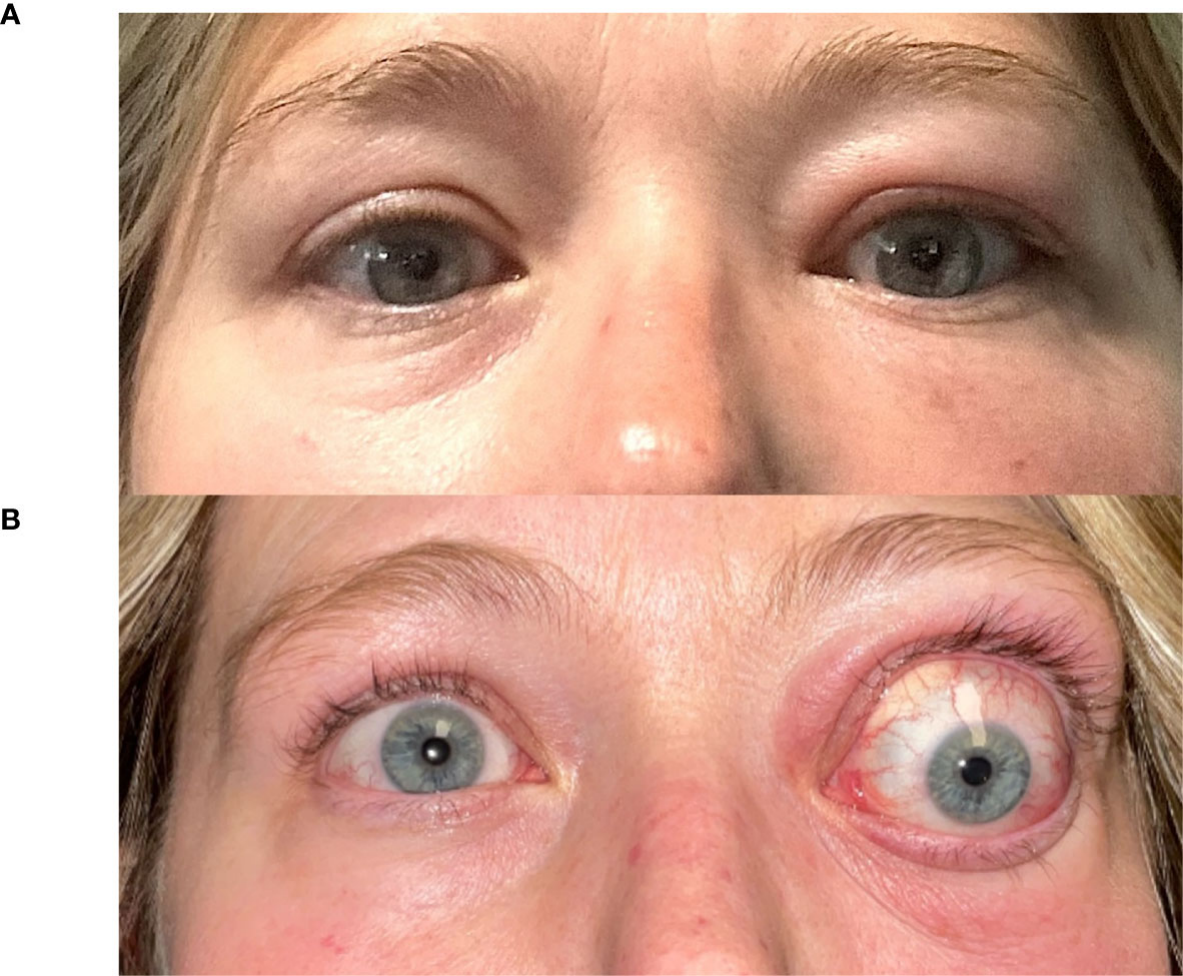
A 43-year-old woman (Case 2) presents with Graves’ disease **(A)**. Following a facial CT scan with iodine contrast, she developed exacerbation of her left-sided periorbital edema, erythema, strabismus and proptosis (7 mm), with new-onset relative afferent pupillary defect **(B)**.

In the authors’ large tertiary referral practices, we have noticed that TED patients exposed to ICM are more likely to present with significant worsening of their orbitopathy within 8-12 weeks of exposure. A case series describing this temporal relationship is provided in **Table 1**, with **Figure 1** (Case 2) demonstrating the dramatic exacerbation of TED symptoms following ICM. All patients had known medical diagnosis of thyroid dysfunction and/or thyroid eye disease, or were undergoing evaluation for suspected TED. None of the patients with known thyroid dysfunction were counseled on the risk of iodine-induced hyperthyroidism or received prophylactic steroids at the time of ICM exposure. Baseline mean thyroid stimulating immunoglobulin (TSI), available in two patients, was 2.17 IU/L (Normal < 0.10 IU/L). TSI at time of presentation following ICM exposure was elevated in the all 4 patients with available bloodwork results. Mean TSI was 10.39 IU/L, but was skewed by one patient with TSI of 39.2 IU/L. Our case series is limited by a small subset of patients; in our experience, many such patients experience an increase in anti-thyroid antibody titers following ICM exposure. For patients who have undergone thyroidectomy (e.g. Case 1), it is unclear whether the orbitopathy flare-up is related to stimulation of a miniscule amount of thyroid tissue versus a direct effect on orbital fibroblasts.

**Table 1:** Case series of patients with TED exacerbation following ICM exposure.

Case	Age, Gender	Previous thyroid therapy	Pre-ICM thyroid status, therapy	Previous TED therapy	Method of ICM exposure	Post-ICM exposure symptoms	Time between ICM exposure and symptom onset (weeks)	Change in CAS	Change in Proptosis (mm)	Subsequent TED intervention
1	62 F	RAI, thyroidectomy, repeat RAI	Hypothyroid, levothyroxine	Intraorbital steroids	CT angiography of the head and neck with and without contrast for evaluation of headache	New-onset symptoms of bilateral ocular surface irritation, tearing, and orbital pressure	8 weeks	+2	0 mm OU	None
2	43 F	None	Hyperthyroid, methimazole	None	CT face with and without contrast for evaluation of the orbits for new onset pain and irritation	Worsening left-sided periorbital edema, erythema, strabismus, proptosis with new-onset relative afferent pupillary defect	4 weeks	+2	0 mm OD, +7 mm OS	Thyroidectomy, followed by orbital decompression and teprotumumab^d^
3	39 F	RAI	Hypothyroid, levothyroxine	None	CT neck with contrast	New onset TED symptoms, with signs of early optic neuropathy	4 weeks	+2	+3 mm OD, +4.5 mm OS^b^	IV methylprednisolone^a,d^
4	50 F	None	Hypothyroid, levothyroxine	None	CT angiography	Worsening left-sided restrictive strabismus and proptosis	2 weeks	+2	+3.5 mm OD, +1 mm OS^c^	IV methylprednisolone^a^, followed by teprotumumab, eventual thyroidectomy and orbital decompression thereafter
5	63 M	None	Euthyroid	Oral Prednisone	CT face with contrast for evaluation of the orbit	New onset bilateral restrictive strabismus	12 weeks	+2	+1.5 mm OD, +2.5 mm OS	IV methylprednisolone^a,d^

TED, Thyroid eye disease; ICM, Iodinated contrast media; CT, Computed tomography; CAS, Clinical Activity Score.

^a^ EUGOGO Protocol (European Group On Graves' Orbitopathy).

^b^ Proptosis comparison measurements obtained 8 months post exposure.

^c^ Proptosis comparison measurements based upon reduction seen at 9 months post medical therapy.

^d^ Active ongoing disease, pending additional intervention.

## Discussion

Our experience reported herein suggests that the presence of, or risk for developing thyroid eye disease is a contraindication for administration of ICM. The worsening orbitopathy observed in our series did not appear to correlate with serum auto-antibody level, although our series is small and auto-antibody testing was not performed universally and consistently. Hence, we speculate that the effects on orbital tissues may be the result of iodine interacting directly with orbital fibroblasts. Given our observations, we recommend updating the teaching materials in the field of Ophthalmology to remove recommendations for ICM administration in evaluation of patients suspected of manifesting thyroid eye disease (6, 7).

For ophthalmologists who take care of patients with thyroid eye disease, knowledge of both the Wolff-Chaikoff and Jod-Basedow phenomenon is important. Patients should be cautioned against the elective use of iodinated contrast media and/or the risk of orbitopathy exacerbation following ICM administration. In evaluating patients with suspected TED, a CT scan without contrast can be used to evaluate extraocular muscle size, the orbital apex and optic nerve relationship, as well as for perioperative planning. If contrast is deemed important, we recommend that magnetic resonance imaging (MRI) with gadolinium contrast would be a safer option.

Practitioners should be aware of other common sources of excess iodine, including iodine-containing medications (e.g. amiodarone, potassium iodide), and dietary supplements (e.g. kelp) (11). Ophthalmologists should also be aware of the risk of systemic absorption of iodine from topical povidone-iodine, which has been reported to result in thyroid dysfunction after application to mucosal surfaces and large surgical wounds (11).

In patients at high risk for developing iodine-induced hyperthyroidism, management remains controversial. Antithyroid medications (methimazole or perchlorate) can be considered prophylactically prior to ICM administration in high-risk patients (5). Extrapolating from studies focused on preventing thyroid orbitopathy activation following radioactive iodine (RAI) and adverse reactions to iodine, patients can also be offered corticosteroid premedication and/or a 4-6 week steroid taper following ICM exposure (17–19). While routine thyroid function testing (i.e. serum TSH) is not generally recommended, TSH monitoring can be obtained 3-4 weeks after ICM exposure in patients at higher risk of thyroid dysfunction, such as elderly patients and patients with unstable cardiovascular disease (5).

If iodine-induced changes are suspected, confirmatory testing may include elevated iodine concentration in a serum or 24-hour urine specimen (2, 5). Depending on the clinical scenario, additional workup can include serum thyroid antibody panel, thyroid ultrasonography, and thyroid scintigraphy (5). For thyrotoxicosis, systemic therapy may include initiation of beta-adrenergic blockers and anti-thyroid medications. The use of oral sodium or potassium perchlorate may further be utilized to inhibit the sodium-iodide symporter, as well as lithium for its thyroid inhibitory effects. Patients may require multispecialty care in conjunction with endocrinology for close monitoring of thyroid function studies, as well as careful observation of TED. In our case series, our patients required additional intervention due to vision-threatening symptoms, including systemic steroids, IGF-1R antagonist therapy, and urgent orbital decompression surgery.

Our case series is limited by a small patient number, its retrospective nature, and lack of consistency in obtaining before/after serum bloodwork. A larger cohort study with analysis of pre- and post- ICM clinical exam findings, thyroid hormone levels, and thyroid autoantibody blood work would be required to further investigate this relationship. Given the variability in thyroid eye disease presentation, a study would need to be adequately powered to account for the diversity in clinical findings. Our goal for this publication is to stimulate provider awareness and encourage future study.

In summary, the totality of published data and our clinical experiences in high-volume TED practices suggest that ICM administration is a risk factor for flare-up of thyroid orbitopathy, whether or not the autoimmune thyroiditis is active. It is unclear whether the mechanism of this effect is indirect through the thyroid tissue or direct through orbital fibroblasts. Future studies are required to better understand the basic and clinical science of this phenomenon.
